# Histomorphological Changes in Breast Lesions: A Retrospective Observational Study

**DOI:** 10.7759/cureus.92801

**Published:** 2025-09-20

**Authors:** Pushpak Chaudhari, Shanu Srivastav, Shilpa Gupta, Naureen Syed, Vivek Sanker, Nilofar M Vora, Lakshmi Venugopal, Rohan Mane

**Affiliations:** 1 General Surgery, King's College Hospital NHS Foundation Trust, London, GBR; 2 Pathology, Terna Medical College, Mumbai, IND; 3 Pathology, Terna Medical College, Navi Mumbai, IND; 4 Pathology, Michigan State University, Michigan, USA; 5 Department of Neurosurgery, Stanford University, California, USA; 6 Internal Medicine, Terna Medical College, Navi Mumbai, IND; 7 Internal Medicine, Government Medical College Kannur, Kannur, IND; 8 Neurological Surgery, University of Nis, Niš, SRB

**Keywords:** benign, biopsy, breast, cancer, fnac, histopathology, malignant, metastasis, microscopy slides

## Abstract

Background

Breast lesions can occur in individuals of all age groups and may include a range of conditions, from benign abnormalities to malignant tumors. Among these, breast cancer is one of the most frequently diagnosed cancers worldwide. Methods used to diagnose breast lesions include histopathology studies and flow cytometry DNA analysis. Some lesions, like cellular fibroadenoma and benign phyllodes tumors, are difficult to differentiate from each other. As their treatment modalities differ from each other, it is important to manage the condition with care.

This study emphasizes the importance of timely diagnosis and treatment of various breast conditions to ensure the best possible outcome for patients.

Objective

To retrospectively analyze the histopathological patterns of breast lesions, along with their demographic and clinical characteristics, among patients presenting to a specialty hospital over a 5-year period.

Methods

A retrospective study was conducted by including the breast biopsy, mastectomy, and Fine Needle Aspiration Cytology (FNAC) reports of all patients who presented with various breast lesions at Terna Speciality Hospital and research center during the period of January 2017 to December 2022. Various demographic and clinical information was gathered through the histopathology request forms and registry. The authors also looked at histopathology slides of various instances that occurred during the research period.

Results

The mean age of diagnosis of various breast lesions was found to be 34.18 (SD +/- 14.92). 83% of lesions presented at less than 50 years of age and 17% at more than 50 years. 97% of the total lesions studied were found among the female population. 98% of all lesions presented with a palpable mass, out of which most (64%) lesions were between 1 and 5 cm in size. The majority of the lesions (45.9%) were right-sided, and only 7.2% were bilateral. Most of the lesions studied presented in the upper outer quadrant.

Most of the benign and inflammatory lesions studied presented in individuals aged 21-30 years, and malignant lesions were found predominantly among individuals aged 61-70 years. No inflammatory lesions were found beyond the age of 60 years, and only one malignant lesion was found below 20 years. Among all lesions studied, benign lesions were most common, found in 406 subjects. 18.5% of all lesions were malignant in nature, among which the majority were invasive breast cancer.

Conclusion

Breast cancer is a complex and heterogeneous disease with varying patterns. Most of the lesions studied were benign in nature, of which the most common was fibroadenoma. The most common malignant lesion in the study was invasive breast cancer - nonspecific type.

Understanding the histopathological patterns of breast lesions is essential for improving the diagnostic and treatment outcomes of this disease.

## Introduction

Breast cancer remains a leading health concern among women worldwide due to its high mortality and morbidity rates. Breast diseases are more prevalent in females compared to males, and the patterns and etiologies of these diseases vary significantly across different countries and ethnic groups. The five-year survival rate for metastatic breast cancer is less than 30%, even with adjuvant chemotherapy [1]. Breast pathology encompasses a wide spectrum, including inflammatory, benign, and malignant disorders. An estimated 200,000 cases of breast disease are diagnosed annually in India and Pakistan [2].

In developed countries, major risk factors for breast cancer include lifestyle modifications, delayed age at marriage, late first childbirth, night-shift work, and the use of hormone replacement therapy [3, 4]. In contrast, in developing countries, high breast cancer incidence and mortality are primarily attributed to a lack of awareness, inadequate screening programs, delayed diagnosis, and limited access to quality medical care [5, 6]. A range of treatment options is available, including surgery, radiotherapy, chemotherapy, endocrine therapy, and immunotherapy [7, 8]. Breast disorders account for approximately 40% of referrals to diagnostic centers among women, with breast masses being the most common presenting complaint. Clinically distinguishing between benign and malignant lesions is often challenging [9].

Although various aspects of breast pathology have been addressed in individual studies, a comprehensive investigation is necessary to provide a holistic understanding of the prevalence and diverse clinical and pathological characteristics of breast lesions. This study focuses on the histomorphology of breast lesions in a developing country context. Our objective is to offer a detailed analysis of the prevalence, clinical presentation, and histopathological features of a wide range of breast conditions - including benign and malignant tumors, fibroadenomas, inflammatory diseases such as granulomatous mastitis (GM), and other abnormalities - based on data from India and Pakistan.

## Materials and methods

Study protocol

The study was approved by the institutional ethical committee (TMCHRC/Surg/2022/IEC Protocol-16/63) to conduct a comparative study. A total sample size of 604 breast histopathology and FNAC reports was chosen from the period between January 2017 and December 2022.

Methodology

This retrospective study included breast biopsy, mastectomy, and FNAC reports of all patients who presented to Terna Speciality Hospital and Research Centre between January 2017 and December 2022. Sample selection was consecutive, and data extraction was performed manually from hospital records. Demographic and clinical information (including age, gender, and relevant clinical details) was obtained from histopathology request forms and the hospital registry.

All available histopathology slides were reviewed by the author, and diagnoses were established in accordance with the World Health Organization (WHO) classification of breast tumors to ensure standardization. Cases with both FNAC and histopathological reports were correlated for diagnostic agreement.

Inclusion criteria

1) All patients who underwent breast FNAC and/or histopathological evaluation (biopsy or mastectomy) at Terna Speciality Hospital between January 2017 and December 2022. 2) Cases with both cytological and histopathological reports available for correlation. 3) Patients of all age groups and genders.

Exclusion criteria

1) Incomplete records lacking either FNAC or histopathology reports. 2) Inadequate or non-diagnostic FNAC samples. 3) Cases involving metastatic lesions to the breast from non-breast primaries.

Statistical analysis

Data were entered and analyzed using Microsoft Excel and SPSS software version 25.0 (IBM Corp., Armonk, NY). Descriptive statistics such as mean, standard deviation, and frequency distributions were used for demographic and clinical variables.

## Results

A total of 604 breast lesions were diagnosed histologically over a period of five years. The mean age at diagnosis was found to be 34.18 years (SD ± 14.92).

The female-to-male ratio of breast lesions was 97:3. A total of 83% of lesions were found in patients under 50 years of age, while 17% were in those over 50. The lesions presented as follows: (310) 51% were right-sided, 251 (41.5%) were left-sided, and (43) 7% were bilateral. In terms of size, 11% of lesions were smaller than 1 cm, 64% ranged from 1 to 5 cm, and 12% were larger than 5 cm. In 98% of cases, the lesions presented with a palpable mass, 4% presented with pain, and 3.3% showed skin involvement. Breast lesions were most commonly found in the upper outer quadrant and least frequently in the lower outer quadrant.

**Figure 1:**
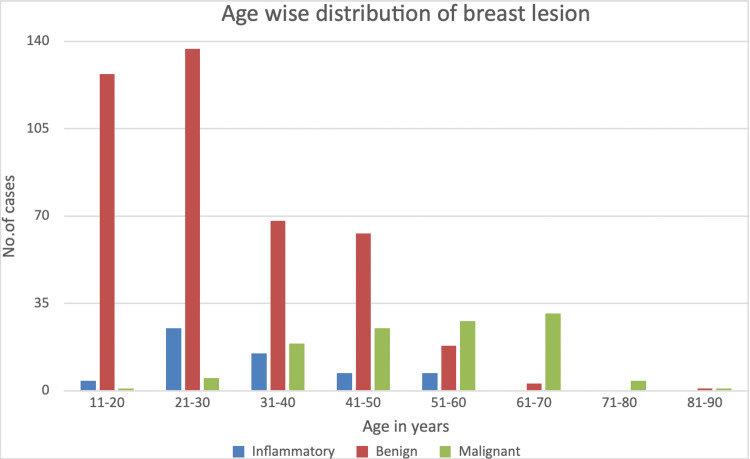
Age-wise distribution of breast lesions

Figure 1 shows the age-wise distribution of breast lesions. Benign and inflammatory lesions were most common in the age group of 21-30, while malignant lesions were most common in the age group of 61-70. A total of 27% of all breast lesions occurred in the 21-30 age group. No inflammatory lesions were observed in individuals older than 60 years. Only one malignant breast lesion was found in a patient younger than 20 years. Benign lesions were the most common in the 11-20 age group.

**Figure 2:**
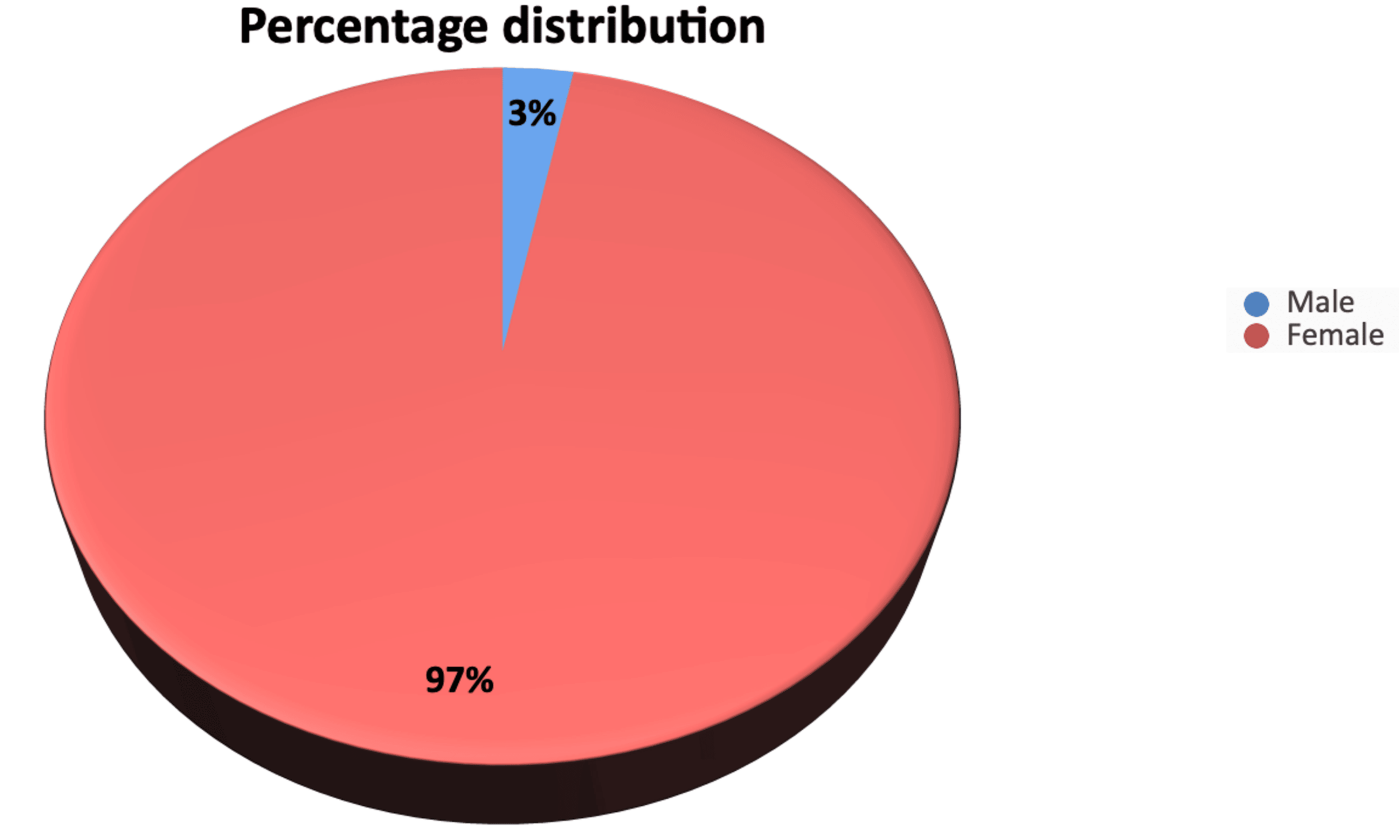
Percentage distribution of male and female cases of breast lesions.

Figure 2 shows the percentage distribution of breast lesions by gender. Females comprised 97% of all breast lesions, while males accounted for 3%.

**Figure 3:**
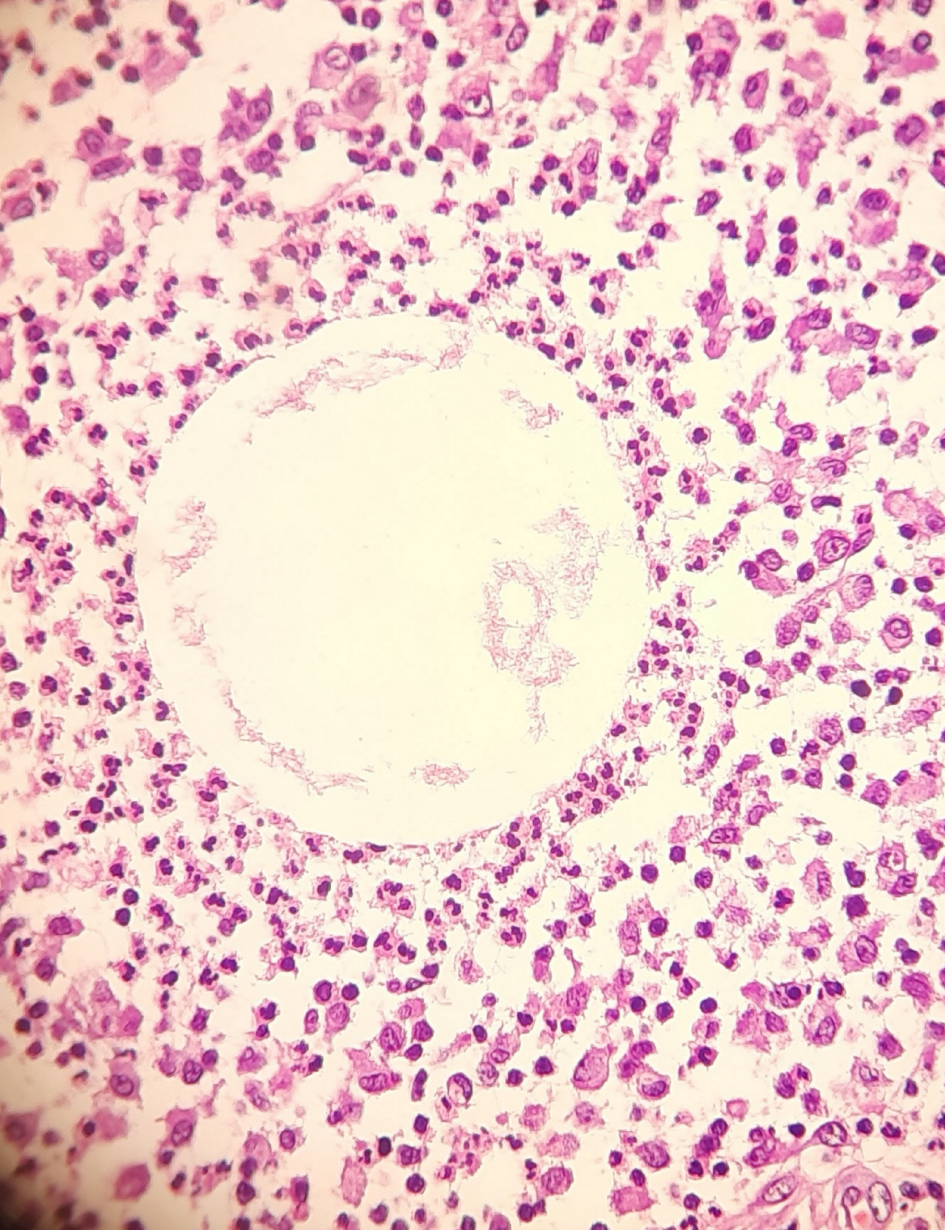
Cystic neutrophilic granulomatous mastitis

Inflammatory breast lesions comprised 9.6% of the total breast lesions. Of these, 1.7% were cases of acute mastitis, 2.5% were cases of granulomatous mastitis, 2% were cases of chronic neutrophilic granulomatous mastitis, and 3.3% were other inflammatory lesions (Table 1). In addition, we observed 2% (12 cases) of cystic neutrophilic granulomatous mastitis (CNGM) (Figure 3).

**Table 1:** Distribution of inflammatory breast lesions

Histopathological Diagnosis Total (n= 58 (9.6%))		Number of Patients	Mean Age (Years)
Acute Mastitis		10 (1.7%)	36
Fat Necrosis		1 (0.16%)	55
Granulomatous Mastitis		15 (2.5%)	33
CNGM		12 (2%)	30
Inflammatory, other n=20 (3.3%)	Antibioma/Breast abscess	2 (0.3%)	29
	Benign cyst	2 (0.3%)	58
	Breast abscess	2 (0.3%)	28
	Chronic granulomatous mastitis	1 (0.16%)	28
	Foreign body granulomatous reaction	1 (0.16%)	24
	Infected granulation tissue with Florid Foreign Body giant cell reaction	1 (0.16%)	42
	Ductal ectasia	8 (1.32%)	34
	Squamous papilloma	1 (0.16%)	22
	Infected keratinous cyst	1 (0.16%)	30
	Others	1 (0.16%)	28

**Figure 4:**
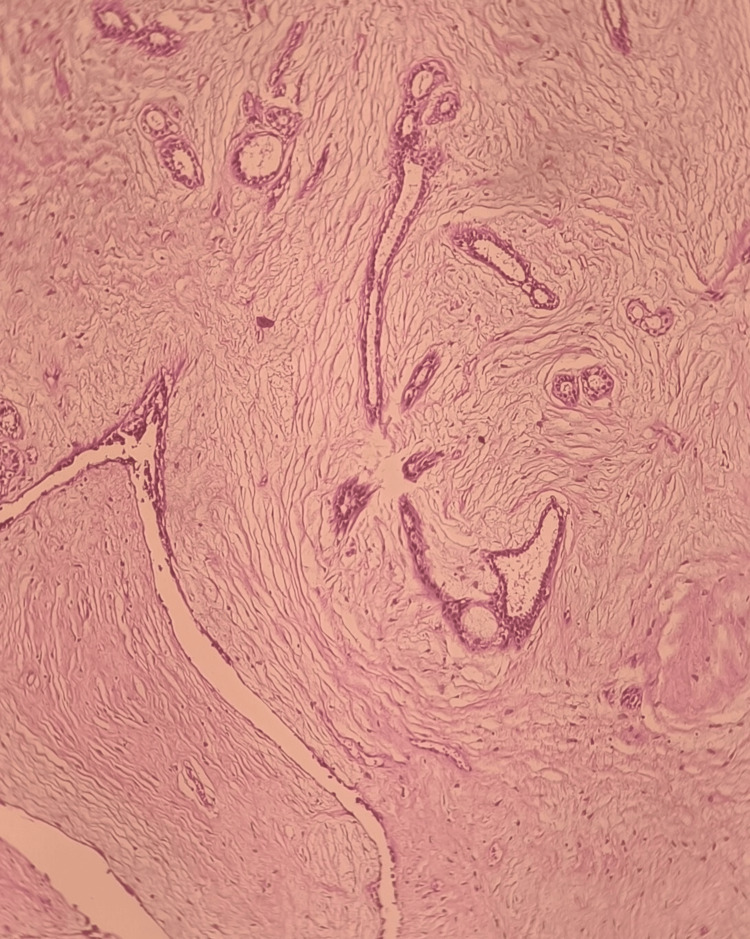
Fibroadenoma

Benign lesions were the most common, accounting for 417 (69%) of all breast lesions. Among these, 278 (46.02%) cases were fibroadenomas (Figure 4), which included classical, juvenile, and fibroadenomas with phyllode-like patterns. Additionally, 11% of lesions were due to fibrocystic changes, 1.6% were phyllodes tumors, and 10.2% were classified as other benign breast lesions (Table 2). 

**Table 2:** Distribution of benign breast lesions

Histopathological Diagnosis Total- n= 417 (69%)		Number of Patients	Mean Age (years)
Fibrocystic changes		67 (11%)	32
Fibroadenoma n=278 (46.02%)	Classical	254 (42.0%)	27
	Juvenile	2 (0.3%)	16
	With phyllodes-like pattern	22 (3.6%)	28
Phyllodes tumor		10 (1.6%)	36
Benign other n= 62 (10.2%)	Adenomyoelithelial adenosis	2 (0.3%)	36
	Complex fibroadenoma	4 (0.6%)	41
	Gynecomastia	12 (2%)	27
	Galactocele	2 (0.3%)	24
	Leiomyoma breast	1 (0.16%)	50
	Lipoma	1 (0.16%)	25
	Myofibroblastoma	1 (0.16%)	20
	Normal ductal hyperplasia	2 (0.3%)	45
	Pseudoangiomatous stromal hyperplasia with usual ductal hyperplasia	1 (0.16%)	48
	Squamous papilloma	1 (0.16%)	16
	Tubular adenoma	8 (1.3%)	21
	Sclerosing adenosis	1 (0.16%)	15
	Intraductal papilloma	3 (0.5%)	27
	Nodular adenosis	1 (0.16%)	40
	Others	22 (3.6%)	36

**Figure 5:**
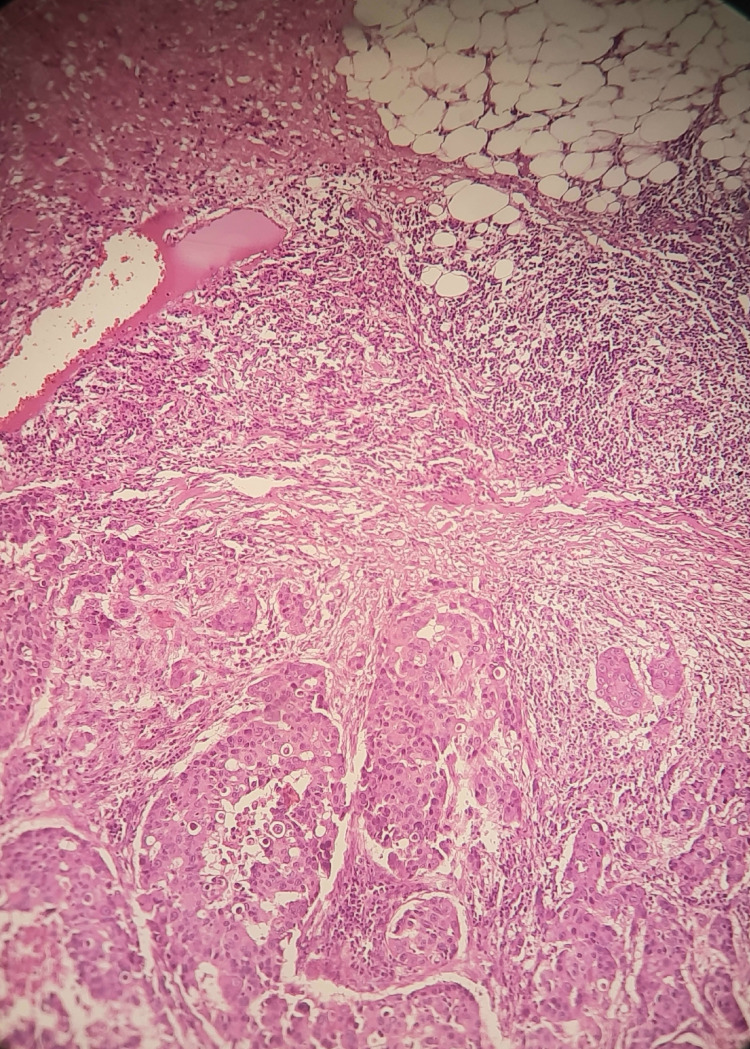
Invasive breast cancer: no special type

Malignant lesions comprised 19.2% of all breast lesions. Of these, 13.6% were invasive breast cancer, 0.16% were lobular carcinoma, and 4.9% were classified as other malignant types (Table 3). Invasive breast cancer - No special type (Figure 5) was the most common, seen in 82 cases (13.6%) among both female and male patients, with a mean age of 53 years.

**Table 3:** Distribution of malignant breast lesions

Histopathological Diagnosis (Total n= 113 (18.7%))		Number of Patients	Mean Age (years)
Invasive breast Ca NST		82 (13.6%)	53
Lobular		1 (0.16%)	31
Malignant Other n=30 (4.96%)	Post lumpectomy, post chemotherapy MRM specimen	1 (0.16%)	49
	Duct papilloma	1 (0.16%)	40
	Poorly differentiated carcinoma	1 (0.16%)	65
	Poorly differentiated epithelial malignancy	1 (0.16%)	66
	Invasive breast CA with neuroendocrine differentiation	1 (0.16%)	69
	Papillary carcinoma	1 (0.16%)	68
	Adenomyoepithelial adenosis with atypia	1 (0.16%)	31
	Mucinous carcinoma	1 (0.16%)	40
	Non-identified malignancies	22 (3.6%)	47

## Discussion

In the last two decades, increased public awareness of breast cancer and the wider application of mammography have dramatically accelerated the incidence of breast diseases. This has made the female breast one of the most commonly biopsied tissues, with an average of 1 out of 6 women worldwide diagnosed [10]. In 2020, breast cancer became the most diagnosed cancer globally, accounting for 1 in 8 cancer diagnoses, with an estimated 2.3 million new cases across both sexes, though with a female predominance [11].

We conducted this study to gain insight into the overall patterns of breast lesions in our region, while considering the variants that mimic breast malignancy and the discrepant patterns of fibroepithelial tumors. Our study reviewed and analyzed 604 cases over five years (2017-2022) of various breast specimens received in different forms: excisional biopsy, tru-cut biopsy, core needle biopsy, simple mastectomy, and modified radical mastectomy, in the Pathology Department at a Tertiary Care Center in Mumbai. After grossing and tissue fixation, microscopic evaluation was conducted. Results showed that the highest number of breast lesions was observed in the 21-30 age group, with 167 (27.6%) cases, followed by the 11-20 group with 132 (21.8%) cases, and the 31-40 group with 102 (16.8%) cases (Figure 1). In total, 493 (81.6%) cases were in patients under 50 years of age. The majority of patients were females, with 586 (97%) presenting with a palpable mass (98%), followed by pain (4%), and a right breast predominance in 299 (49.5%) cases. Our study is limited to lesion location, with 41 (6.7%) cases involving the upper outer quadrant.

Benign breast diseases were the most common breast lesions observed in our study, with 417 (69%) cases, which is consistent with previous studies (Table 1, Table 4). Based on histopathology, fibroadenoma was the most common breast lesion, found in 278 (46%) cases, including 254 (42%) classical cases. Higher rates of fibroadenoma were reported by Aslam et al. (2013) (71%), Padmom et al. (2020) (57%), and Poojasaree et al. (2021) (54.2%), while lower rates were observed by Olu-Eddo and Ugiagbe (2011) (43.1%) [12-15]. The peak incidence of fibroadenoma in our patients occurred in the 3rd (27.6%) and 2nd (21.8%) decades of life (mean age 27 years). A wide age range (12-90 years) was observed, suggesting a hormonally triggered mechanism due to excessive estrogen circulation [10,12,13,16-20]. Interestingly, two (0.3%) juvenile fibroadenoma cases were seen in our study, presenting as rapidly enlarging painless palpable masses of up to 5 cm in size in 16-year-old adolescents with stromal and epithelial hypercellularity. All findings were consistent with studies by Basara and Balci (2021) and Lerwill et al. (2022) [20-21]. Another notable finding was the presence of two (0.3%) complex fibroadenoma cases, which may increase the threefold risk of breast cancer development [20,21]. The presence of duct epithelial hyperplasia, sclerosing adenosis, and papillary apocrine changes is characteristic of complex fibroadenoma [20,21].

**Table 4:** Malignant breast lesions in different studies. DE: duct ectasia; FCD: fibrocystic disease; FA: fibroadenoma; PT: phyllodes tumor; GM: gynecomastia; IDC: infiltrating ductal carcinoma

Study	Region	IBC n/mean	Lobular carcinoma n/mean	Medullary carcinoma n/mean	Mucinous carcinoma n/mean
Kale et al., n=194 [18]	India	104 (53.6%)	2 (1.03%)	---	1 (0.51%)
Aslam et al. N=254 [12]	Pakistan	30 (11.8%)	--	--	--
Present study n=604	India	82 (13.6%)	1 (0.16%)	-	1 (0.16%)

Benign biphasic breast lesions, such as phyllodes tumors, were seen in 1.6% of all patients (10 cases), with a peak incidence during the premenopausal age (mean age of 36.5 years) [20,21]. Histologically, phyllodes tumors present as an intraductal growth of intralobular stroma with a leaf-like epithelial pattern [12,13,19,21]. A lower frequency was noted by Aslam et al. (2013) (1.2%) and Sulhyan et al. (2017) (1.24%), while a higher frequency was reported by Poojasree et al. (2021) (10%) [12,14,22].

Fibrocystic changes were the second most common benign breast lesions found in our study, with 67 (11%) cases (mean age 32 years). Patients presented with a palpable mass (100%) and pain (4%), which aligns with previous studies (Table 4) [12,13,19,22-24]. These changes are due to an imbalance of female sex hormones during ovulation and just before menstruation, causing fluid retention in breast cells, resulting in nodules and cysts throughout the breast. They usually regress in the postmenopausal phase [10].

In our study, we observed eight rare cases of tubular adenoma (1.3%), consistent with current statistics, involving young females of reproductive age (average age 21 years) [13,18,24,20,25]. Additionally, three cases of lactating adenoma (0.5%) were noted [13,18,20]. Interestingly, a single case of sclerosing adenosis (0.16%), a lobular lesion with increased fibrous tissue and glandular cells, was observed, which carries a twofold risk of breast cancer [24,16]. Additionally, two extremely rare cases of adenomyoepithelial adenosis (AMEA) (0.3%) were noted in our study, occurring during the perimenopausal age (36 years), making it a high-risk factor for developing invasive ductal carcinoma.

Inflammatory breast lesions accounted for 9.6% (58 cases) of our study, with a peak incidence in the 3rd decade of life (mean age 33.1 years). Higher frequencies were previously reported by Rezaianzadeh et al. (2014) (39%), while Olu-Eddo et al. (2011) (5.9%) reported lower frequencies [9,15]. Granulomatous mastitis was the most common inflammatory lesion (2.5%) (15 cases), mostly occurring in the premenopausal age, consistent with previously published studies [26]. High levels of estrogen and/or progesterone (including exogenous use), elevated prolactin, and infectious factors, as well as non-infectious factors such as autoimmune diseases like sarcoidosis, foreign substances like silicone, paraffin, or trauma, have been presumed to contribute to the pathogenesis of granulomatous mastitis [26]. In addition, we observed 2% (12 cases) of cystic neutrophilic granulomatous mastitis (CNGM), predominantly in females with a peak incidence in the 4th decade (mean age 30 years). These lesions have characteristic morphological features, including granulomas comprised of epithelioid histiocytes and giant cells with central lipid vacuoles rimmed by neutrophils. Occasional rod-shaped, gram-positive bacilli are also seen within the vacuoles [27]. Other inflammatory lesions seen in our study included acute mastitis in 1.7% (10 cases) of females (mean age 36 years) presenting as sub-centimeter painful masses, mostly affecting the right breast. Furthermore, 1.32% (8 cases) of duct ectasia were observed, mostly in the 5th decade (mean age 34 years) in females. The most striking finding was the presence of bilateral ductal ectasia with infiltrative ductal carcinoma in two of our patients.

Similar to previous studies, our study reports breast diseases as more prevalent among females than males. However, the most common male breast disease seen in our study was gynecomastia (2%) (12 cases) in the 3rd decade, with an average age of 27 years. This finding is consistent with reports by Qadri et al. (2019) (6.7%), Yogalakshmi and Kavitha (2019) (5%), Jamal (2001) (3.1%), Albasri (2014) (3.1%), and Aslam et al. (2013) (3.9%) [10,12,19,24,28]. Gynecomastia results from an imbalance between estrogen and androgen hormones, but it is idiopathic in 40% of cases [10-12]. It has also been observed physiologically in neonates, at puberty, and in old age, but may occur secondary to medications, systemic disorders, paraneoplastic hormone production by the germ cells, or mechanical trauma. The most common presentation is mastalgia.

Breast cancer is the most diagnosed cancer in the world and the leading cause of cancer mortality in women, with a five-year survival rate for metastatic breast cancer of less than 30%. The origin of breast cancer is multifactorial and complex. In developed countries, the main risk factors include prolonged exposure to estrogen, reproductive factors, modified lifestyle, and environmental and genetic factors. In developing countries, however, breast cancer incidence is mainly attributed to a lack of awareness of the disease, inappropriate screening programs, delayed diagnosis, and insufficient medical facilities. Previous studies have shown that breast cancer is heterogeneous in origin and caused by a variety of genetic alterations, which lead to vastly different disease manifestations in individual patients [29]. This “intertumoral heterogeneity” largely affects patient prognosis and treatment options.

Our study identified 114 malignant breast lesions (19.2%). We observed a higher frequency of malignant lesions compared to previous studies reported by Amin et al. (2009) (21.3%) and Sulhyan et al. (2017) (16.6%) [22,23]. Most of these reports are from Pakistan, Saudi Arabia, and India. We also observed a lower frequency reported from India and Pakistan by Padmom et al. (2020) (11.7%) and Aslam et al. (2013) (11%) [12,13]. Our patients initially presented with breast lumps. Additionally, skin ulceration was noted in 14 cases, nipple discharge in 2 cases, and a single case of nipple retraction, consistent with previous studies [14]. Some interesting findings of our study included right-sided breast lesions in 56 (49%) cases (n=114), left-sided lesions in 52 (45.6%) cases (n=114), and bilateral lesions in 6 (5.2%) cases, with limited data regarding quadrant involvement [14]. In contrast, left breast involvement has been reported in other studies [30]. Another finding in our study is the presentation of advanced disease, reflected by tumor size: >5 cm in 32 (28%) cases (n=114) and 1-5 cm in 46 (40%) cases (n=114). This late presentation of the disease is influenced by socioeconomic status, level of education, marital status, and healthcare access. Although this feature is associated with poor prognosis, our patients further underwent axillary lymph node dissection in 40 (35%) cases (n=114), but we have limited data on tumor grading.

Many factors, such as educational level, economic status, environmental conditions, food habits, ethnic and geographical variations, globalization, and cultural practices, influence breast cancer incidence globally, including in both developing and developed countries [17]. Reports have shown that breast cancer incidence in urban India is most common in the age group of 40-49 years, while in rural India, it occurs in the 65-69 age group [30]. The mean age of presentation in our study was 52.2 years, which is lower than that reported by Ambroise et al. (2011) (53.8 years) [31]. Leong et al. compared the peak age of cancer between the Asian and Western woman populations, which peaks at 40-50 years in the Asian population and 60-70 years in the Western population [32]. We also observed a younger age of presentation, with a mean age of 48.5 years, as reported by Jamal (2001) from Saudi Arabia, 48.9 years by Rezaianzadeh et al. (2014) from Iran, and 45.6 years by Aslam et al. (2013) from Pakistan [9,12,28]. During the International Surgical Week for Breast Surgery in 2007, it was argued that the peak age for breast cancer in developing countries is between 40 and 50 years, whereas in developed countries it is between 60 and 70 years [33]. Insufficient early diagnosis and effective treatment remain crucial factors for the increased breast cancer mortality in developing countries [34]. A key finding in our study was that 67 (58.7%) of the cases (n=114) were in patients over 50 years of age, while 47 cases (41.2%) (n=114) were in those under 50 years old. The peak age of distribution was found in the 7th decade, between 61-65 years, followed by the 6th decade, between 56 and 60 years. However, Sulhyan et al. (2017) observed the maximum incidence during the 6th decade of life [22].

Invasive breast cancer - No special type (IBC-NST) is the most common variety of invasive breast cancer (75-80%). In our study, IBC-NST was the most common, seen in 82 cases (13.6%) among both female and male patients, with a mean age of 53 years. As listed in Table 5, this was reported by Kale et al. (2023) from India at 53.6%, all of which are higher than our study [18]. However, Aslam et al. (2013) from Pakistan reported 11%, which is lower than our study [12]. The prognosis of IBC-NST depends on factors such as the patient’s age, tumor size, lymph node status, histologic grade, and lymphovascular invasion, which is associated with a higher risk of local and distant recurrence. A higher percentage of breast carcinoma with nodal metastasis has been reported in Asian and Indian studies compared to the West [30]. As noted earlier, 78 cases (68%) (n=114) presented with tumor sizes larger than 1 cm in our study.

**Table 5:** Frequency distribution of various benign breast lesions in different studies. DE: duct ectasia; FCD: fibrocystic disease; FA: fibroadenoma; PT: phyllodes tumor; GM: gynecomastia; IDC: infiltrating ductal carcinoma

Study	Region	Mastitis n/mean	DE n/mean	FCD n/mean	FA n/mean	PT n/mean	GM n/mean	IDC n/mean
Amin et al., n=969 [23]	KSA	142 (14.6%)	109 (11.2%)	120 (12.3%)	311 (32.0%)	3 (0.3%)		163 (16.8%)
Aslam et al., n=254 [12]	Pakistan	14 (5.6%)	---	3 (1.2%)	181 (71.3%)	3 (1.2%)	10 (3.9%)	30 (11.8%)
Albasri, n=1005 [24]	KSA	60 (5.9%)	14 (23%)	141 (23.4%)	267 (44.03%)	16 (2.6%)	19 (3.1%)	__
Sulhyan et al., n=161 [22]	India	14 (8.6%)	7 (4.34%)	6 (3.72%)	60 (37.2%)	2 (1.24%)	4 (2.48%)	43 (26.7%)
Yogalakshmi and Kavitha, n=120 [19]	India	7 (5.8%)	__	15 (12.5%)	45 (37.5%)	1 (0.83%)	6 (5%)	17 (14.1%)
Padmom et al., n=867 [13]	India	26 (2.9%)		88 (10%)	496 (57%)	5 (0.5%)		92 (10.6%)
Poojasree et al., n=140 [14]	India	___	___	25 (17.8%)	76 (54.2%)	14 (10%)	___	___
Olu-Eddu and Ugiagbe, n=2575 [15]	Nigeria	___	44 (2.5%)	444 (23.8%)	43.1 %	34 (1.8%)	39 (2.1%)	
Present study	India	26 (4.3%)	8 (1.32%)	67 (11%)	278 (46%)	10 (1.6%)	12 (2%)	82 (13.6%)

A rare finding in our study was four cases of male breast cancer (0.66%), which presented as chest nodules during the 7th decade of life (60-65 years), with a mean age of 64 years, which aligns with previous studies [35]. It has been reported that breast cancer in men is commonly associated with poor prognosis and mortality due to a lack of disease knowledge, poor socioeconomic status, delays in seeking medical help, and delayed disease detection in males. Geographic variation has also been noted, as breast cancer represents less than 1% of all breast cancers in the USA but a higher proportion in Africa [35]. It is also increasing worldwide due to factors such as increased longevity, obesity, testicular diseases, Klinefelter's syndrome, drugs, exogenous hormones, and germline mutations of BRCA2 [36,28]. The prognosis of male breast cancer is similar to that of female breast cancer, depending on the tumor stage, nodal status, and molecular subtype. Male breast cancer patients are associated with an overall poorer outcome compared to females, with a reduced 5-year survival rate (82.8% vs 88.5%) and a 43% higher risk of death in men than in women [37]. Apart from primary lesions, metastatic lesions to the breast in men have also been reported [36].

Despite multiple treatment options, including surgery, radiotherapy, chemotherapy, endocrine therapy, and immunotherapy, breast cancer incidence and mortality continue to increase globally [17]. The lack of multimodal treatment facilities, overcrowded treatment centers, and financial constraints contribute to treatment delays. As mentioned earlier, late presentation with local advancement is more common in developing countries [17,28]. Established screening programs in developed countries have contributed to the early detection of breast cancer at the preinvasive stage and earlier stages [31]. Therefore, strategies should be implemented to develop and improve ongoing breast disease screening programs, with clear objectives of raising awareness, education, and careful diagnostic approaches to prevent patient anxiety and delays in diagnosis.

This study adds additional information to the international research literature conducted at a tertiary hospital in Mumbai, although it may not represent the entire nation.

Limitations of the study

This study has several limitations. It was conducted at a single tertiary care center in Mumbai, limiting its generalizability to broader or rural populations. As a retrospective study, it is subject to selection and reporting biases, with incomplete or inconsistent data in some cases. Additionally, there was limited information on tumor grading, molecular subtypes, and prognostic factors like hormone receptor status, which could have provided a more comprehensive understanding of breast cancer prognosis. The lack of longitudinal follow-up data prevents assessment of patient outcomes, recurrence rates, or survival. Furthermore, the study did not incorporate radiological data to correlate with histopathological findings, and the predominantly female cohort may not fully represent male breast cancer patterns.

## Conclusions

This study underscores the diverse histopathological spectrum of breast lesions observed over a five-year period at a tertiary care center in Mumbai. Benign lesions were significantly more prevalent than malignant ones, with fibroadenoma emerging as the most common benign entity, predominantly affecting younger females. Invasive breast carcinoma of no special type (IBC-NST) was the most frequent malignant lesion, primarily presenting in older women. The study also documented rare lesions such as juvenile fibroadenoma, adenomyoepithelial adenosis, and male breast carcinoma, highlighting the diagnostic complexity and spectrum of breast pathology.

These findings emphasize the critical role of histopathological examination in the accurate diagnosis and classification of breast lesions, which is essential for guiding appropriate clinical management. The notable incidence of large tumor size at presentation and age-related distribution patterns point toward the continued need for early detection initiatives, increased public awareness, and better access to diagnostic services - particularly in developing regions.

Although limited to a single institution, this study contributes valuable data to the regional literature and supports the implementation of robust screening protocols to improve patient outcomes. Further multi-centric research involving larger and more diverse populations is recommended to validate these findings and to better understand the evolving trends in breast disease pathology.
